# Reactive Oxygen Species Distribution Involved in Stipe Gradient Elongation in the Mushroom *Flammulina filiformis*

**DOI:** 10.3390/cells11121896

**Published:** 2022-06-11

**Authors:** Junjie Yan, Julia Chekanova, Yuanyuan Liu, Bingcheng Gan, Ying Long, Xing Han, Zongjun Tong, Juan Miao, Lingdan Lian, Baogui Xie, Fang Liu

**Affiliations:** 1 Mycological Research Center, College of Life Sciences, Fujian Agriculture and Forestry University, Fuzhou 350002, China; yanjunjie@caas.cn (J.Y.); chekanovaj@fafu.edu.cn (J.C.); 2200514015@fafu.edu.cn (Y.L.); longying@caas.cn (Y.L.); hanxing@caas.cn (X.H.); tongzongjun@saas.sh.cn (Z.T.); miaoj@biomarker.com.cn (J.M.); lingdanlian@scau.edu.cn (L.L.); 2 Institute of Urban Agriculture, Chinese Academy of Agricultural Sciences, Chengdu 610213, China; ganbingcheng@caas.cn; 3 The Key Laboratory of Extreme-Environmental Microbiology (Liaoning Province), College of Plant Protection, Shenyang Agricultural University, Shenyang 110866, China

**Keywords:** *Flammulina filiformis*, fruiting-body development, reactive oxygen species signaling, mushroom stipe elongation, NADPH oxidase, superoxide dismutase

## Abstract

The mushroom stipe raises the pileus above the substrate into a suitable position for dispersing spores. The stipe elongates at different speeds along its length, with the rate of elongation decreasing in a gradient from the top to the base. However, the molecular mechanisms underlying stipe gradient elongation are largely unknown. Here, we used the model basidiomycete mushroom *Flammulina filiformis* to investigate the mechanism of mushroom stipe elongation and the role of reactive oxygen species (ROS) signaling in this process. Our results show that O_2_^−^ and H_2_O_2_ exhibit opposite gradient distributions in the stipe, with higher O_2_^−^ levels in the elongation region (ER), and higher H_2_O_2_ levels in the stable region (SR). Moreover, NADPH-oxidase-encoding genes are up-regulated in the ER, have a function in producing O_2_^−^, and positively regulate stipe elongation. Genes encoding manganese superoxide dismutase (MnSOD) are up-regulated in the SR, have a function in producing H_2_O_2,_ and negatively regulate stipe elongation. Altogether, our data demonstrate that ROS (O_2_^−^/H_2_O_2_) redistribution mediated by NADPH oxidase and MnSODs is linked to the gradient elongation of the *F. filiformis* stipe.

## 1. Introduction

As a remarkable feature in the development of basidiomycete fruiting bodies, the stipe elongation raises the pileus into a position suitable for spore dispersal and thus ensures the propagation of the species [1,2]. For cultivated edible mushrooms, the stipe elongation rate is important for efficient growth; in addition, stipe length determines product quality by producing the classic cap-and-stem mushroom shape [3]. Moreover, stipe elongation is primarily based on cell elongation, providing an excellent model for studying hyphal cell elongation [4]. Therefore, the mechanisms governing stipe elongation are of great interest in the field of mushroom biology [5,6,7,8,9]. 

Stipe elongation is non-uniform: the elongation rate decreases in a gradient from the apex to the base of the stipe, as demonstrated in basidiomycete mushrooms [10]. Niu et al. [11] suggested that the gradient stipe elongation rate in *Coprinopsis cinerea* may result from gradients of cell wall composition, architecture, and thickness from the apical to the basal regions. Our previous results also showed that expression of the genes encoding the enzymes S-adenosylmethionine-dependent methyltransferase and cytochrome c peroxidase was higher in the apical region than in the basal region of *Flammulina velutipes* stipes [12,13,14]. However, the molecular and signaling mechanisms of stipe gradient elongation remain unknown.

Reactive oxygen species (ROS), especially superoxide anion (O_2_^−^) and hydrogen peroxide (H_2_O_2_), are key signaling molecules in cell proliferation, development, tissue differentiation, and environmental responses in many organisms [15,16,17]. Cellular O_2_^−^ is mainly produced by mitochondrial electron transport chains and NADPH oxidase, and is converted to H_2_O_2_ by superoxide dismutases (SODs) [18,19]. In plants, variations in ROS levels (H_2_O_2_, O_2_^−^, and •OH) affect cell wall loosening during cell elongation and cross-link formation, and thus mediate cell wall elongation [20,21]. Moreover, ROS act as key signaling molecules that regulate leaf, root, and fiber elongation, as shown in several plant species [22,23,24,25]. Multiple studies in the model plant *Arabidopsis thaliana* have demonstrated that the balance of O_2_^−^ and H_2_O_2_ controls root elongation and determines the transition between cell proliferation and differentiation [26,27]. 

In fungi, the NADPH-oxidase-derived ROS signaling pathway is critical for polar growth of hyphae, fruiting body formation, and development [28,29]. The polar growth proteins Bem1 and Cdc24 are components of the NADPH oxidase complex and are required for morphogenesis and growth of fungal hyphae [30]. The NADPH oxidase subunits NoxA, NoxB, and NoxR have been suggested to be essential for ROS generation, hyphal branching, and fruiting body development in the medicinal Basidiomycete *Ganoderma lucidum* [31]. We also found that SOD genes were differentially expressed during fruiting body development in the edible mushroom *Volvariella volvacea* [32]. However, the role of the ROS signaling pathway in mushroom stipe elongation is still unknown.

Several mushroom species are used to study stipe elongation. Among them, the commercially important cultivated mushroom *Flammulina filiformis* (previously known as *F. velutipes*), which has a very long stipe and small genome size, represents a superb model system for studying the molecular and signaling mechanisms involved in regulating stipe elongation [33,34,35]. Here, we examined the mechanism of stipe elongation in *F. filiformis* and found that O_2_^−^ and H_2_O_2_ play an important role in this process. Our findings suggest that the interplay between O_2_^−^ and H_2_O_2_ and their redistribution may act as the primary determinants of the gradient elongation of the *F. filiformis* stipe. These findings provide insight into the mechanisms controlling gradient elongation of the stipe in mushrooms. 

## 2. Materials and Methods

### 2.1. Strains and Growth Conditions

A commercial dikaryotic strain of *Flammulina filiformis*, Fv01 (mated to monokaryotic Fv01-10 and Fv01-N strains, with a white fruiting body), was obtained from the Fujian Edible Fungi Germplasm Resource Collection Center of China. The mycelium was maintained on potato dextrose agar medium (200 g/L potato; 20 g/L glucose; 20 g/L agar) at 25 °C. The fruiting bodies were grown on substrate medium (58% cottonseed hulls, 20% sawdust, 20% wheat bran, 1% calcium sulphate dihydrate, and 1% sucrose (all percentages are *w*/*w*), then adjusted to a total moisture content of 60%) and cultivated in bottles according to the method in Park et al. [35]. The temperature was maintained at 10 °C during stipe elongation.

### 2.2. Stipe Elongation Measurement and Cell Length Detection

The stipe was marked in 1.5 cm intervals starting at the apex. Then, the marked fruiting bodies were grown continuously on the substrate medium, and the change in length of each section was measured over time.

Approximately 3 mm long fresh segments of the stipe region were cut using a scalpel and collected; the surface mycelium was examined under an Olympus microscope BX51 (Olympus Corporation, Tokyo, Japan), the micrographs were taken, and the lengths of stipe mycelium cell were measured by Image J 1.51j8 software.

### 2.3. ROS Detection

Nitro blue tetrazolium chloride (NBT, Amresco, Fountain Parkway, Solon, OH, USA) and 3, 3′-diaminobenzidine (DAB) were used for histochemical detection of O_2_^−^ and H_2_O_2_, respectively, according to the method in Kumar et al. [36]. The NBT working solution was 0.05% (*w*/*v*), and staining was performed at room temperature with light for 20 min. The DAB working solution was 1 mg/mL, and staining was performed at room temperature without light for 8 h.

The fluorescent probes dihydroethidium (DHE) and 2′, 7′-dichlorodihydrofluorescein diacetate (DCF) were used to detect O_2_^−^ and H_2_O_2_, respectively [37]. The DHE working solution was 10 μM, and staining was performed at room temperature without light for 30 min. The DCF working solution was 10 μM and staining was performed at room temperature without light for 20 min. A Leica SP8 confocal microscope (Leica, Heidelberg, Germany) was used to detect the fluorescence. For DHE staining, dye excitation was set at 300 nm and emitted light was detected at 610 nm. For DCF staining, dye excitation was set at 488 nm and emitted light was detected at 550 nm. The images were processed with Leica Confocal Software.

A commercial oxygen free radical assay kit (Suzhou Comin Biotechnology, Suzhou, China) was used to determine the O_2_^−^ content. O_2_^−^ reacts with hydroxylamine hydrochloride to generate NO_2_^−^, and NO_2_^−^ forms a red azo compound in the presence of p-aminobenzene sulfonic acid and α-naphthylamine. This compound was measured at 530 nm and the absorbance was used to calculate the O_2_^−^ content (oxidation of hydroxylamine method). A commercial hydrogen peroxide assay kit (Suzhou Comin Biotechnology, Suzhou, China) was used to determine the H_2_O_2_ content. H_2_O_2_ bound to titanium sulfate forms a yellow titanium peroxide complex, which was measured at 415 nm and the absorbance was used to calculate the H_2_O_2_ content (titanium sulfate colorimetric method).

### 2.4. Gene Identification and Sequence Analysis

The amino acid sequences of the genes encoding NADPH oxidase subunits and SOD family proteins of filamentous fungi were downloaded from GenBank and used for BLAST queries with the 14,270 amino acid sequences of the *F. filiformis* strain Fv01-10 genome (Accession: PRJNA769814) using BLASTP (e-value set as 1 × 10^−2^). The gene sequences were verified by Sanger sequencing after PCR amplification; the PCR primers were designed using Primer Premier 6.0 and synthesized by Sangon Biotech Co., Ltd. (Shanghai, China) (Table S1). Gene structure analysis was performed according to the method of Yan et al. [32] by using strand-specific RNA-seq data of mixed samples of Fv01 mycelium and fruiting bodies [14]. The gene schematic diagram was drawn by the Gene Structure Display Server (GSDS 2.0) (http://gsds.cbi.pku.edu.cn/index.php, accessed on 8/20/2019). The accurate amino acid sequences were submitted to Conserved Domain Database (https://www.ncbi.nlm.nih.gov/cdd, accessed on 20 August 2019), the ProtParam tool (https://web.expasy.org/protparam/, accessed on 20 August 2019), TMHMM Server v. 1.0 (https://embnet.vital-it.ch/software/TMPRED_form.html, accessed on 20 August 2019), ProtComp 9.0 (http://linux1.softberry.com/berry.phtml?topic=protcompan&group=programs&subgroup=proloc, accessed on 20 August 2019), and SignalP-5.0 Server (http://www.cbs.dtu.dk/services/SignalP/, accessed on 20 August 2019) for bioinformatics.

### 2.5. Gene Expression Analysis

A commercial E.Z.N.A. Plant RNA kit (Omega Bio-Tek, Norcross, GA, USA) was used to isolate the total RNA of samples. TransScript One-Step gDNA Removal and cDNA Synthesis SuperMix (TransGen Biotech, Beijing, China) and TransScript All-in-One First-Strand cDNA Synthesis SuperMix for qPCR (One Step gDNA Removal) kits (TransGen Biotech, Beijing, China) were used for cDNA synthesis. Gene expression was detected by quantitative real-time PCR using TransStart Top Green qPCR SuperMix (TransGen Biotech, Beijing, China). The genes encoding glyceraldehyde-3-dehydrogenase (FfGAPDH) and Ras family Small GTPase (FfRas) were used as the reference genes [38]. The qPCR primers were designed using Primer Premier 6.0 and synthesized by Sangon Biotech Co., Ltd. (Shanghai, China) (Table S2). The relative expression levels were calculated by the 2^−ΔΔCt^ method [39].

### 2.6. SOD Enzyme Activity Measurement

SOD activity was detected according to the method of Li et al. [40]. Inhibition of the photochemical reduction of NBT was monitored, and one unit of SOD activity was defined as the amount of enzyme required for 50% inhibition of the reduction of NBT monitored at 560 nm.

### 2.7. DPI Treatment

The fruiting body elongation stage was selected for DPI treatment. The stipes were marked at 1.5 cm intervals starting at the junction with the pileus; the marked fruiting bodies were either untreated or soaked in water (two controls), or subjected to 50 μM DPI treatment for 30 min to inhibit NADPH. After that, the fruiting bodies were placed vertically, supported by damp paper, and grown at 10 °C in the dark for 72 h before the level of O_2_^−^ was determined by staining.

### 2.8. Overexpression and RNA Interference Constructs and Fungal Transformation

The binary transformation vector pBHg-eGFP (NCBI accession no. MZ420392), stored in the Mycological Research Center of Fujian Agriculture and Forestry University, Fuzhou, China, was used to construct the RNA interference (RNAi) and overexpression (OE) plasmids. The pBHg-eGFP plasmid included the *hygromycin B phosphotransferase* (*Hyg*) gene driven by the *Glyceraldehyde-3-phosphate dehydrogenase* (*GAPDH*) promoter from *Agaricus bisporus*, and the gene encoding enhanced green fluorescent protein (*eGFP*) driven by the *GAPDH* promoter from *F. filiformis*. For construction of the OE plasmid, the *eGFP* sequence of the pBHg-eGFP plasmid was replaced by the target gene sequence. For the RNAi plasmid, the *eGFP* sequence was replaced by the hairpin RNA (hpRNA) construct. In detail, the 4th intron (58 bp) of *FfNoxA* in strain Fv01-10 was selected as the loop of the hpRNA construct. The 4th exon (295 bp) of *FfNoxA* was selected as the arm of the hpRNA for pBHg-FfNoxA-RNAi plasmid, the coding sequences (without the ATG region) of *FfMnSOD1* were selected as the arms of the hpRNA for pBHg-FfMnSOD1-RNAi plasmid, and coding sequences (without the ATG region) of *FfMnSOD2* were selected as the arm of the hpRNA for pBHg-FfMnSOD2-RNAi plasmid. All plasmids were verified by sequencing, the schematic representation of OE and RNAi constructs were drawn by SnapGene 1.1.3 (Figure S1), and the sequences were submitted to the NCBI database (MZ374061–MZ374064).

The plasmids were transformed into *F. filiformis* dikaryotic strain Fv01 by the *Agrobacterium tumefaciens*–mediated transformation method according to Wu et al. [41], with minor modifications. First, each recombinant plasmid was introduced into *A. tumefaciens* strain GV3101, and transformation of modified mycelia pellets was performed. Transformed colonies were selected on CYM medium (0.2% yeast extract, 0.2% tryptone, 1% maltose, 2% glucose, and 2% agar; all percentages are *w*/*v*) containing 50 μg/mL hygromycin B four times. The T-DNA insertion site(s) of transformants were verified by whole-genome resequencing according to Chou et al. [42]. Gene expression was normalized to the transcript level of the reference gene *FfGAPDH* and calculated relative to the transcript level in the wild type (WT) according to the 2^−ΔΔCT^ method [39].

### 2.9. Phenotype Analysis of Transformants

To investigate the phenotype changes of the transformants in the mycelium stage, 6 mm diameter modified mycelia pellets of the WT (strain Fv01) and transformants were inoculated onto a new potato dextrose agar plate and incubated in the dark at 25 °C for 6 days. The O_2_^−^ and H_2_O_2_ concentrations were measured by NBT and TMB histochemical detection [43], respectively. The microscopic images were recorded with an Olympus BX51 microscope. 

To assess the relation between gene function and stipe elongation, the fruiting bodies of the WT and transformants were cultivated under the same conditions as in Park et al. [35]. Fruiting bodies with 5 cm long stipes were selected, the area 1.5 cm away from the stipe apex was marked, the region from the apex to the mark was measured over 6 days, and the elongation rates were calculated. To assess the relation between gene function and stipe elongation ability, the fruiting bodies were cultivated until stipe elongation ceased, and the longest 7 fruiting bodies of each bottle were selected for total stipe length measurement.

### 2.10. Statistical Analysis

The statistical analysis was performed by Graphpad Prism 6.0 (Graphpad software, San Diego, CA, USA). The mean ± S.E.M. was determined for each treatment group in the individual experiments. Statistical significance between samples was investigated by Student’s *t*-test or Tukey’s multiple comparisons test.

## 3. Results

### 3.1. Gradient Elongation of the F. filiformis Stipe

*F. filiformis* fruiting body development is divided into primordium, differentiation, elongation, and maturation stages, each accompanied by distinct morphological changes (Figure S2). The mushroom stipe starts elongating at the differentiation stage; elongation increases at the elongation stage, and then slows down substantially at the maturation stage. 

**Figure 1:**
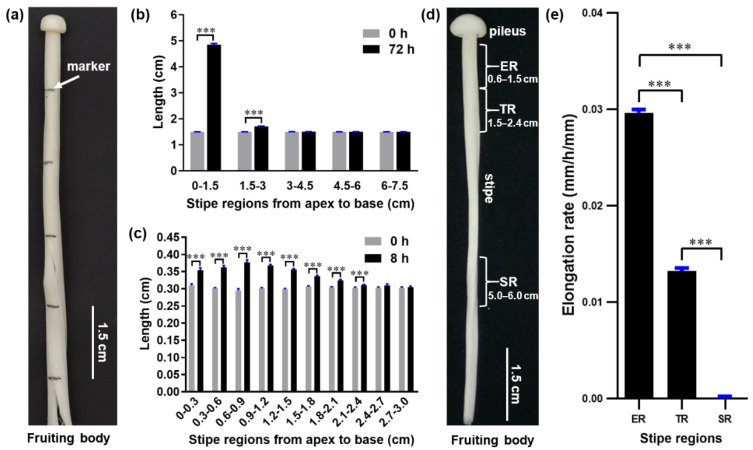
The distribution of elongation along the length of the *F. filiformis* stipe. (**a**) Marking of the stipe. (**b**) Length of different stipe regions after 72 h growth (*t*-test, *** *p* < 0.001, *n* = 50). (**c**) Stipe elongation characteristics of the apical 3-cm stipe regions (*t*-test, *** *p* < 0.001, *n* = 20). (**d**) Different stipe regions: ER, elongation region; TR, transition region; SR, stable region. (**e**) Elongation rates of different stipe regions over 12 h of growth (Tukey’s multiple comparisons test, *** *p* < 0.001, *n* = 20).

To investigate the elongation pattern of *F. filiformis* stipes and determine which regions elongate the fastest, we used mushrooms in the elongation stage, when the stipe reached approximately 8.5 cm. Then, we subdivided the stipes from the apex to the base into 1.5 cm regions, labeled them with a marker (Figure 1a), and allowed the fruiting bodies to grow for 72 h to reach the near-maturation stage. Examination of individual regions revealed that the apical 0–1.5 cm region was the most extended, elongating by an additional ~3.3 cm, and that the 1.5–3.0 cm region exhibited only ~0.2 cm of elongation. By contrast, the 3.0–4.5 cm, 4.6–6.0 cm, and 6.0–7.5 cm regions did not elongate (Figure 1b).

To more precisely determine the main elongating region within the apical 0–1.5 cm and 1.5–3.0 cm regions, we subdivided these regions into 0.3 cm intervals, labeled them, and measured their length at 0 and 8 h of growth. We found that all 0.3 cm intervals in the top 0–1.5 cm region elongated rapidly, with the 0.6–0.9 cm segment extending the most, while the 0.3–0.6 cm and 0.9–1.2 cm segments elongated more slowly (Figure 1c). The elongation speed slowed dramatically in the region located below 1.5 cm, and the stipe stopped extending in the region below 2.4 cm.

These measurements show that the 0.6–1.5 cm region is the fastest elongating segment of the stipe; therefore, we designated this as the elongation region (ER) and designated the slowly elongating region (1.5–2.4 cm) as the transition region (TR) (Figure 1d). The segment with no elongation (5–6 cm) was designated the stable region (SR) (Figure 1d). These regions were chosen for further studies to investigate the molecular mechanism of gradient elongation of the stipe. To confirm our observations, we allowed mushrooms of the same stage to grow for 12 h and determined the elongation speed of the ER, TR, and SR. Our results confirm that the elongation rate of the ER was 2.2 times faster than that of the TR, while the SR had no further elongation (Figure 1e).

### 3.2. O_2_^−^ and H_2_O_2_ Contents Show Opposite Gradient Distributions on the F. filiformis Stipe

**Figure 2:**
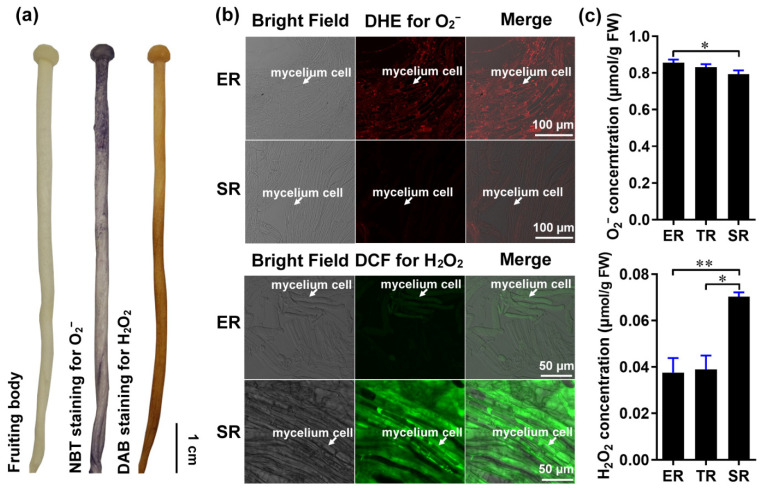
The distribution of ROS (O_2_^−^ and H_2_O_2_) in the stipe of *F. filiformis*. (**a**) Histochemical staining with NBT to detect O_2_^−^ (purple) and DAB to detect H_2_O_2_ (yellowish-brown). (**b**) Cellular-level fluorescent probe detection with DHE for O_2_^−^ (red fluorescence) and DCF for H_2_O_2_ (green fluorescence) in ER and SR region cells (the mycelium cells were marked by white arrows). (**c**) Measurement of the O_2_^−^ (*n* = 5) and H_2_O_2_ (*n* = 3) concentration using spectrophotometry (paired *t*-test, * *p* < 0.05, ** *p* < 0.01).

Gradient root elongation in *A. thaliana* relies on the distribution of O_2_^−^ and H_2_O_2_ [26]. To investigate whether these ROS are involved in controlling stipe gradient elongation in mushrooms, we determined the distributions of O_2_^−^ and H_2_O_2_ in the stipes using nitro blue tetrazolium chloride (NBT) and 3,3′-diaminobenzidine (DAB) histochemical staining, respectively. Our results reveal the gradient distribution of O_2_^−^ and H_2_O_2_ in the *F. filiformis* stipes, with O_2_^−^ accumulating in the ER and H_2_O_2_ accumulating in the non-elongating SR (Figure 2a).

We also examined O_2_^−^ and H_2_O_2_ distributions at the cellular resolution by laser scanning confocal microscopy using the fluorescent dyes dihydroethidium (DHE) and 2′, 7′-dichlorodihydrofluorescein diacetate (DCF), respectively. Consistent with the histochemical staining, we found that the ER contains a high concentration of O_2_^−^ and a low concentration of H_2_O_2_, while the SR contains a high concentration of H_2_O_2_ and a low concentration of O_2_^−^ (Figure 2b). To quantitatively confirm the distributions of O_2_^−^ and H_2_O_2_ in the stipe, we measured their concentrations by spectrophotometry. The O_2_^−^ and H_2_O_2_ concentrations differed significantly between the ER and SR, with O_2_^−^ gradually decreasing from the ER to the SR, and H_2_O_2_ increasing from the ER to the SR (Figure 2c). 

These results reveal a differential gradient distribution of O_2_^−^ and H_2_O_2_ in different regions of the stipe and suggest a potential role of ROS signaling in controlling *F. filiformis* stipe elongation.

### 3.3. Genes Encoding NADPH Oxidase and MnSODs Are Differentially Expressed in Different Regions of F. filiformis Stipes

**Figure 3:**
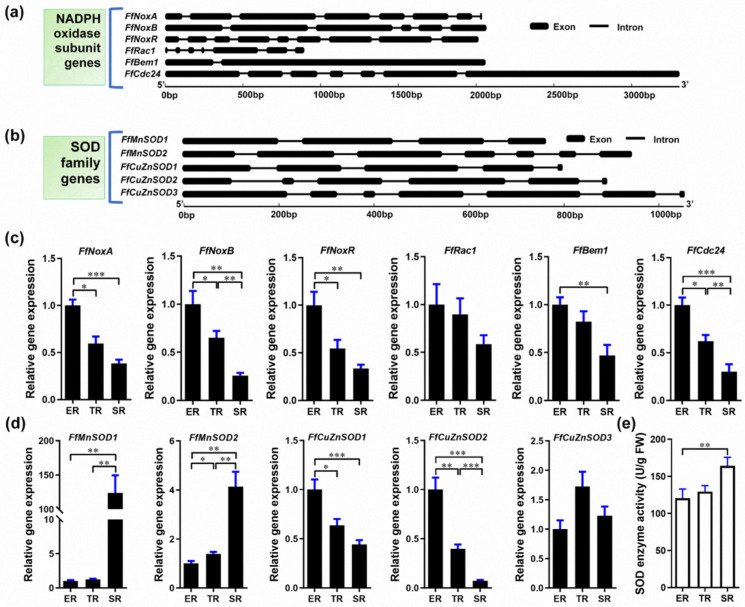
Identification of *Flammulina* NADPH and SOD homologs and their expression in the ER (elongation region), TR (transition region), and SR (stable region). (**a**) Gene structure of *Flammulina* NADPH oxidase subunit homologs. (**b**) Gene structure of *Flammulina* SOD family homologs. (**c**) Relative expression of genes encoding NADPH oxidase subunits in different stipe regions (*n* = 6). (**d**) Relative expression of SOD family genes (*n* = 6). (**e**) SOD enzyme activity (*n* = 5) in different stipe regions. The significance levels were calculated by paired *t*-test and marked as follows: * *p* < 0.05, ** *p* < 0.01, *** *p* < 0.001.

O_2_^−^ is generated in eukaryotic cells via various mechanisms and NADPH oxidase is among the major O_2_^−^ sources [44,45]. In fungi, NADPH oxidase is a multi-subunit membrane-bound enzyme complex. SOD is the main enzyme that catalyzes the dismutation of O_2_^−^ into H_2_O_2_ [19]. To investigate the O_2_^−^ and H_2_O_2_ gradient distribution in the *F. filiformis* stipe, we examined the expression of the genes encoding NADPH oxidase subunits and SODs. To this end, we sequenced and assembled the *F. filiformis* genome and used it to bioinformatically identify *F. filiformis* homologs of NADPH oxidase subunits and SOD family genes. We identified six *F. filiformis* NADPH oxidase subunit homologs: FfNoxA, FfNoxB, FfNoxR, FfRac1, FfBem1, and FfCdc24. The gene structures are shown in Figure 3a and the GenBank IDs are MN661156–MN661161, respectively. Bioinformatics analysis of the NADPH oxidase subunits showed that all proteins contain complete conserved domains, suggesting that the *F. filiformis* NADPH oxidase is a functional complex (Table S3). In addition, FfNoxA and FfNoxB subunits were predicted to be plasma membrane proteins with 7 transmembrane helices, consistent with NADPH oxidase localization in the cell membrane (Table S3). We performed qRT-PCR to investigate the expression patterns of the NADPH subunit genes and found that all six genes mimicked the pattern of O_2_^−^ localization, which was highest in the ER and lowest in the SR (Figure 3c). Pearson’s correlation coefficient analysis showed that the expression patterns of all six genes were positively correlated with the stipe elongation rate and O_2_^−^ production (Table S4).

We then investigated the *F. filiformis* SOD homologs. We found five genes encoding two SODs of the manganese (Mn) type (FfMnSOD1 and FfMnSOD2, GenBank IDs AYG96714 and AYG96715, respectively), and three SODs of the copper (Cu)/zinc (Zn) type (FfCuZnSOD1, FfCuZnSOD2, and FfCuZnSOD3, GenBank IDs AYG96711–AYG96713). The gene structures are shown in Figure 3b. Bioinformatics analysis of these genes shows that all the encoded proteins contain complete conserved domains, suggesting that the *F. filiformis* genome encodes five functional SOD enzymes (Table S3). Subcellular localization analysis predicted that the two FfMnSODs are located in mitochondria with a high prediction score, and the three FfCuZnSODs are most likely located in the cytoplasm. Moreover, we found a secretory Sec/SPI signal peptide in the FfCuZnSOD2 sequence, which indicates that FfCuZnSOD2 might be secreted, unlike the MnSODs. Furthermore, the gene expression patterns showed that both *FfMnSOD* genes had the highest expression in the SR and the lowest expression in the ER, which show a positive correlation with SOD enzyme activity (Figure 3d,e). Of note, *FfMnSOD1* expression was 124 times and 99 times higher in the SR than in the ER and TR, respectively (Figure 3d). The expression patterns of *FfCuZnSOD1* and *FfCuZnSOD2* are negatively correlated with SOD enzyme activity, while *FfCuZnSOD3* shows no significant differences among the different stipe regions (Figure 3d,e). The gene expression data combined with the subcellular localization and signal peptide prediction suggest that the CuZnSODs might play a role in stipe elongation.

Altogether, these results suggest that the specific expression of NADPH oxidase genes in the ER and MnSOD genes in the SR could result in the differential distribution of O_2_^−^ and H_2_O_2_ in different regions of *F. filiformis* stipes.

### 3.4. NADPH-Oxidase-Derived O_2_^−^ Positively Regulates Stipe Elongation

**Figure 4:**
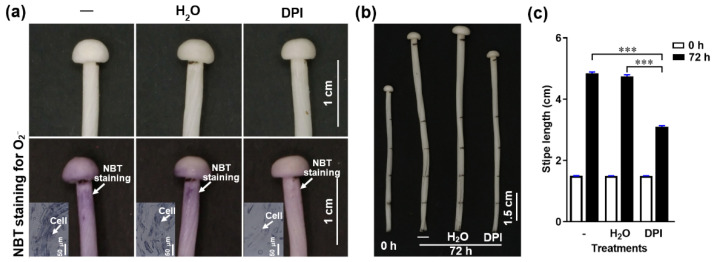
The effect of the NADPH oxidase inhibitor (DPI) on O_2_^−^ production and stipe elongation. (**a**) The O_2_^−^ detected by NBT histochemical staining after different treatments. The O_2_^−^ is visualized as purple deposits with NBT (marked by white arrows). The inserts in the bottom row panels correspond to the histochemical staining on a cellular level. (**b**) Stipe elongation phenotype upon different treatments after 0 and 72 h cultivation.—corresponds to normal growth without any treatment, H_2_O means the stipe was soaked in water for 30 min, DPI means the stipe was soaked in 50 μM DPI for 30 min. The junction between the pileus and stipe and each 1.5 cm region from the stipe apex to the base were labelled before treatment. (**c**) Stipe elongation of the ER measured at 0 and 72 h of cultivation after treatment.—corresponds to normal growth without any treatment, H_2_O indicates water treatment for 30 min, DPI indicates 50 μM DPI for 30 min. The significance levels were calculated by paired t-test and marked as follows: *** *p* < 0.001, *n* = 50.

To determine if NADPH-oxidase-derived O_2_^−^ regulates stipe elongation, we treated *F. filiformis* stipes with the NADPH oxidase inhibitor diphenyleneiodonium chloride (DPI) and measured the O_2_^−^ distribution and stipe elongation. We found that O_2_^−^ concentration and stipe elongation were significantly inhibited after pre-incubation in DPI for about 30 min (Figure 4), which indicates that the NADPH oxidase and the O_2_^−^ it generates are important factors in stipe elongation. 

**Figure 5:**
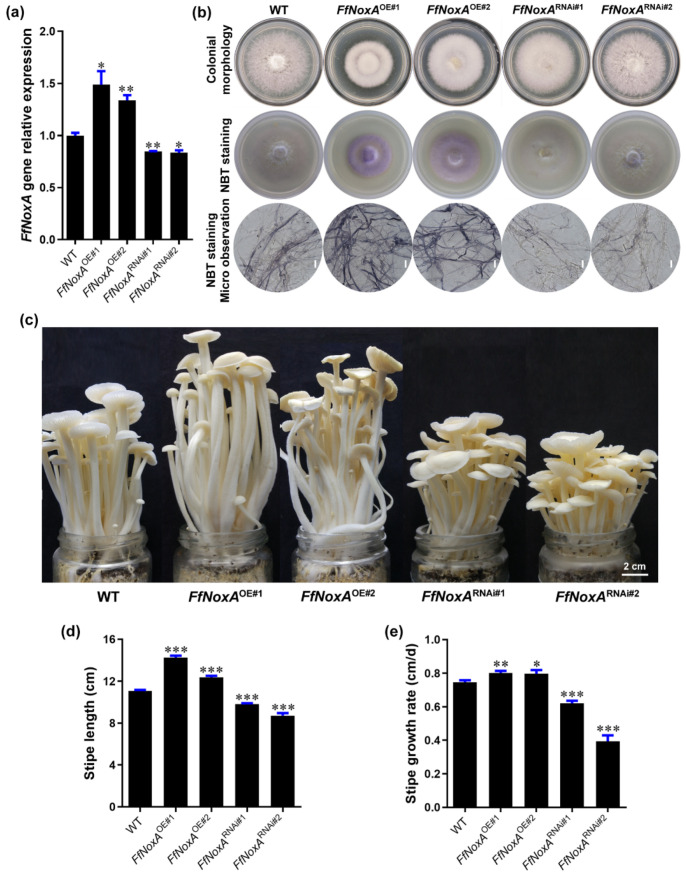
Gene expression, ROS detection, and stipe elongation phenotype in the wild type (WT) and *FfNoxA* overexpression and RNAi lines. (**a**) The relative expression of *FfNoxA* in the WT, overexpression lines, and RNAi lines. (**b**) Colony morphology and histochemical staining of cells with NBT for O_2_^−^ detection (purple) in *FfNoxA* overexpression and RNAi lines (Bar = 20 μm). (**c**) The phenotype of fruiting bodies at the maturation stage (Bar = 2 cm). (**d**) The total stipe length at the maturation stage (*n* = 35). (**e**) Growth rate measurement of the 0–1.5 cm stipe region in *FfNoxA* overexpression and RNAi lines (*n* = 25). The significance levels were calculated by paired *t*-test compared with the WT sample, * *p* < 0.05, ** *p* < 0.01, *** *p* < 0.001.

*FfNoxA* encodes an NADPH oxidase subunit with core transmembrane and catalytic domains [30]. Therefore, to confirm the function of NADPH oxidase in controlling the O_2_^−^ concentration and stipe elongation, we created *FfNoxA* overexpression and knock-down RNAi lines. We obtained five independent overexpression transformants and four independent RNAi transformants. Two of the overexpression transformants (*FfNoxA*^OE#1^ and *FfNoxA*^OE#2^) and two of the RNAi transformants (*FfNoxA*^RNAi#1^ and *FfNoxA*^RNAi#2^) with a verified single T-DNA insertion site for each transformant were selected for further study (Table S5). The qRT-PCR results confirm that the transcript abundance of *FfNoxA* in the overexpression lines was significantly increased, and that in RNAi lines it was significantly reduced, compared with the WT mushrooms (Figure 5a).

To confirm that the transgenic lines differed in O_2_^−^ production relative to the WT strain, we detected the endogenous O_2_^−^ level via NBT staining in transgenic and WT mycelium. In agreement with the role of NoxA in O_2_^−^ production, as a catalytic subunit of NADPH oxidase [31], *FfNoxA* overexpression increased O_2_^−^ levels, while RNAi of *FfNoxA* reduced O_2_^−^ levels compared with WT mycelium (Figure 5b), supporting the idea that NADPH oxidase mediates O_2_^−^ production.

To examine whether *FfNoxA* is involved in stipe elongation, we cultivated *FfNoxA* overexpression and knock-down lines, grew their fruiting bodies until stipe elongation ceased, and compared them to the WT mushrooms. We found that the transgenic lines displayed dramatic differences in their appearance and the rates of growth compared to the WT. The measurements of the total length of mature fruiting bodies showed that the *FfNoxA*^OE#1^ and *FfNoxA*^OE#2^ overexpression lines were 28.4% and 11.5% longer than the WT, respectively (Figure 5c,d). The total length of the RNAi lines *FfNoxA*^RNAi#1^ and *FfNoxA*^RNAi#2^ were 11.7% and 21.4% shorter than the WT (Figure 5c,d). 

Additionally, to confirm the function of *FfNoxA* in stipe elongation, we compared the growth rate of the main elongating stipe region (0–1.5 cm) in the overexpression and knock-down lines relative to the WT mushrooms. The elongation rates of the two overexpression lines were significantly higher than in the WT (0.80 ± 0.01 cm/day and 0.80 ± 0.02 cm/day for *FfNoxA*^OE#1^ and *FfNoxA*^OE#2^, respectively) (Figure 5e). The elongation rates of the RNAi lines were 0.62 ± 0.02 cm/day and 0.39 ± 0.03 cm/day for *FfNoxA*^RNAi#1^ and *FfNoxA*^RNAi#2^, respectively, and were significantly lower than in the WT (0.75 ± 0.01 cm/day). These data and the observations that inhibition of *FfNoxA* decreased O_2_^−^ production strongly indicate that *FfNoxA* and its associated O_2_^−^ production are required for stipe elongation in *F. filiformis*.

### 3.5. MnSODs Convert O_2_^−^ to H_2_O_2_ to Negatively Regulate Stipe Elongation

Since the Mn-type SOD genes *FfMnSOD1* and *FfMnSOD2* were highly expressed in the SR and reduced in the ER, particularly *FfMnSOD1* (Figure 3c), we examined the function of *FfMnSOD1* and *FfMnSOD2* in controlling the levels of O_2_^−^ and H_2_O_2_ as well as mushroom growth. To this end, we created *FfMnSOD1* and *FfMnSOD2* overexpression and knock-down RNAi lines. We obtained three independent *FfMnSOD1* overexpression lines and four independent *FfMnSOD1* RNAi lines as well as three independent *FfMnSOD2* overexpression lines and two independent *FfMnSOD2* RNAi lines. 

**Figure 6:**
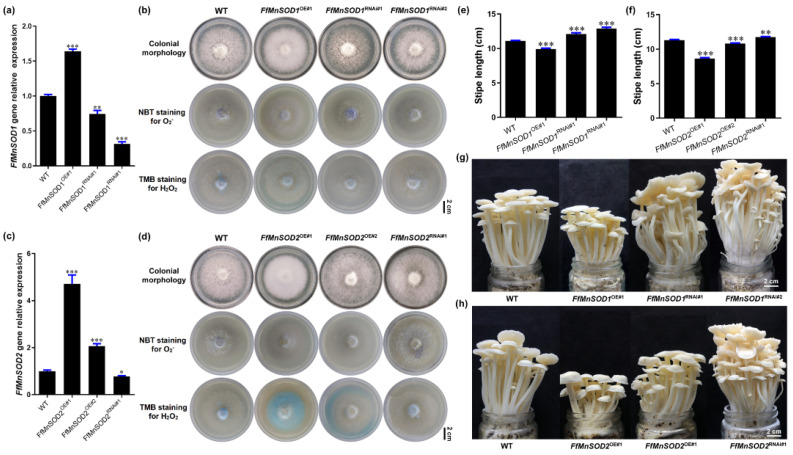
Gene expression, ROS detection, and stipe elongation phenotype in the wild type (WT) and *FfMnSOD1* and *FfMnSOD2* overexpression and RNAi lines. (**a**) The relative gene expression in the WT, *FfMnSOD1* overexpression lines, and RNAi lines. (**b**) Colony morphology, and histochemical staining with NBT for O_2_^−^ (purple) and TMB for H_2_O_2_ (blue) detection in WT, *FfMnSOD1* overexpression lines, and RNAi lines (Bar = 2 cm). (**c**). The relative gene expression in the WT, *FfMnSOD2* overexpression lines, and RNAi lines. (**d**) Colony morphology, and histochemical staining with NBT for O_2_^−^ (purple) and TMB for H_2_O_2_ (blue) detection in the WT, *FfMnSOD2* overexpression lines, and RNAi lines (Bar = 2 cm). (**e**) The total stipe length at the maturation stage in WT, *FfMnSOD1* overexpression lines, and RNAi lines (*n* = 35). (**f**) The total stipe length at the maturation stage in the WT, *FfMnSOD2* overexpression lines, and RNAi lines (*n* = 35). (**g**) The phenotype of fruiting bodies of the WT, *FfMnSOD1* overexpression lines, and RNAi lines at the maturation stage (Bar = 2 cm). (**h**) The phenotype of fruiting bodies of the WT, *FfMnSOD2* overexpression lines, and RNAi lines at the maturation stage (Bar = 2 cm). The significance levels were calculated by paired t-test compared with the WT sample, * *p* < 0.05, ** *p* < 0.01, *** *p* < 0.001.

To confirm that the transformants each contained a single T-DNA insertion site, we conducted genome resequencing and T-DNA insertion analysis (Tables S6 and S7). We selected one overexpression line and two RNAi lines of *FfMnSOD1* (*FfMnSOD1*^OE#1^, *FfMnSOD1*^RNAi#1^, and *FfMnSOD1*^RNAi#2^) for further analysis. For *FfMnSOD2*, we selected two overexpression lines and one RNAi line (*FfMnSOD2*^OE#1^, *FfMnSOD2*^OE#2^, and *FfMnSOD2*^RNAi#1^). The qRT-PCR results confirm that, in comparison with the WT, the transcript abundance of the target genes in all overexpression lines was significantly increased, and that in the RNAi lines it was significantly reduced (Figure 6a,c).

We also examined the endogenous levels of O_2_^−^ and H_2_O_2_ in these transgenic lines by detecting O_2_^−^ with NBT staining and H_2_O_2_ with 3,3′,5,5′-tetramethylbenzidine (TMB) staining. In agreement with the role of SODs in O_2_^−^ conversion to H_2_O_2_ [18], both FfMnSOD1 and *FfMnSOD2* overexpression lines had decreased O_2_^−^ levels and increased H_2_O_2_ levels, while the RNAi lines had increased O_2_^−^ levels and decreased H_2_O_2_ levels, compared with the WT mycelium (Figure 6b,d). 

To examine the growth of the *FfMnSOD1* and *FfMnSOD2* overexpression and knock-down RNAi lines, we allowed them to grow until stipe elongation ceased and compared the transgenic lines to the WT mushrooms (Figure 6e–h). The transgenic lines displayed significant differences in their appearance and growth rates. The stipe length of mature fruiting bodies was shorter in the *FfMnSOD1* and *FfMnSOD2* overexpression lines than in the WT. Considering that these lines had lower O_2_^−^ levels in the stipes, these results strongly indicate the importance of O_2_^−^ in stipe elongation. As expected, the stipes were longer in the *FfMnSOD1* and *FfMnSOD2* RNAi lines than in the WT (Figure 6e–h). 

These results suggest a critical role of *FfMnSOD1* and *FfMnSOD2* in controlling the conversion of O_2_^−^ to H_2_O_2_, and a negative role of H_2_O_2_ in stipe elongation. Altogether, our results suggest that the interplay between O_2_^−^ and H_2_O_2_ signaling is important in modulating elongation of different regions of the stipe in *F. filiformis*.

## 4. Discussion

Several studies have shown that the stipe elongates faster in the apical region in mushrooms such as *F. velutipes* [33,46], *C. cinerea* [47], and *A. bisporus* [2]. However, the ERs differ among species and the mechanisms of gradient stipe elongation are unknown. For *Flammulina*, the area of elongation is uncertain. For example, Fang et al. [33] showed that the cell wall extension activity is located exclusively in the 1.0 cm apical region for mushrooms with 3.0 cm long stipes, while Kern et al. [46] found that elongation is restricted to a 0.2 to 0.3 cm apical zone of the stipe. Although these studies show conflicting results, they indicate that the area of elongation could be specific to the growth stage. In our research, we used mushrooms in the elongation stage, when the fruiting bodies reach about 8.5 cm, thus making it easier to study the elongation mechanism. We found that the top 1.5 cm of the stipe elongates quickly, with the 0.6–0.9 cm segment being the most extended, while the regions below 3.0 cm do not elongate. These results also indicate that the stipe ER could be determined by multiple factors, including species, growth stage, and environmental conditions.

Although high levels of ROS (O_2_^−^ and H_2_O_2_) are harmful to cells, ROS are important signaling molecules necessary for many biological processes [16]. In animal cells, O_2_^−^ signaling regulates cell proliferation and H_2_O_2_ signaling supports quiescence [48]. In plants, O_2_^−^ and H_2_O_2_ have antagonistic roles in stem cell regulation and the ROS balance defines stem cell fate [44]. It has also been suggested that the distribution of O_2_^−^ and H_2_O_2_ is involved in gradient root elongation in *A. thaliana* [26]. In eukaryotic cells, mitochondria and NADPH oxidase are two major sources of O_2_^−^ [44,45]. It has also been suggested that O_2_^−^ generated from membrane-localized NADPH oxidase plays a key role in fungal cellular differentiation and development [28]. Gradient stipe elongation is an important process in macro fungi; therefore, we investigated the mechanism of this process and examined the role of ROS. In this study, we found that the differential expression of genes encoding NADPH oxidase, which produces O_2_^−^ in the elongation region, and MnSODs, which convert O_2_^−^ to H_2_O_2_ in the stable region, could lead to the respective distributions of O_2_^−^ and H_2_O_2_ that result in gradient stipe elongation. Our findings provide insight into the mechanisms of mushroom stipe elongation and indicate that O_2_^−^ and H_2_O_2_ have opposing functions in controlling fungi cell states. Our results suggest a conserved function of ROS in regulating cell states during development across the animal, plant, and fungal kingdoms.

How do O_2_^−^ and H_2_O_2_ participate in this process? A recent study reported that lateral cell extension is the main mechanism of stipe elongation in mushrooms, and that the extent of cell wall loosening controls the ability of cells to elongate [9]. In plants, the wall-loosening reaction controlling cell elongation is affected by the production of reactive oxygen intermediates [20,21,49]. For example, in maize (*Zea mays*), O_2_^−^ serves as a precursor for hydroxyl radical (•OH), which loosens cell walls and thus facilitates cell elongation [50]. Here, we demonstrated that the O_2_^−^ generated by NADPH oxidase plays an important role in stipe elongation, and the membrane-bound catalytic subunit of NADPH, NoxA, positively controls stipe elongation. At the same time, H_2_O_2_ mediates cell-wall stiffening and decreased cell-wall extensibility in maize coleoptiles [51]. In accord with this, we found that two genes encoding mitochondria-localized *FfMnSODs* were significantly up-regulated in the SR of the stipe, which also showed very high SOD enzyme activity, suggesting that H_2_O_2_ enrichment in the SR and inhibited stipe elongation could be due to mitochondria-produced O_2_^−^ converted to H_2_O_2_ by *FfMnSODs*. 

However, we also found that genes encoding a different kind of SOD, *FfCuZnSOD1* and *FfCuZnSOD2*, had significantly higher expression in the ER, which is contrary to the ROS distribution between the elongation region (ER) and the stable region (SR). Therefore, what could be the potential role of the *FfCuZnSOD* genes in the elongating region? We hypothesize that FfCuZnSODs and FfMnSODs play different roles and may be important in cell-wall loosening for elongation due to their distinct cellular localization and properties. Such precedents have been demonstrated in plants. For example, in cotton (*Gossypium hirsutum*), the extracellular Cu/Zn superoxide dismutase (GhCSD3) specifically translocates to cell walls, despite the absence of a signal peptide. There, it produces high levels of H_2_O_2_ and reacts with metal ions, producing •OH in elongating fiber cell walls via the Fenton reaction and loosening cell walls for elongation [52]. Our analysis also showed that FfCuZnSOD2 contains a secretory Sec/SPI signal peptide, making it potentially able to translocate to cell walls, which suggests that the high expression of *FfCuZnSOD2* in the elongating region may play a positive role in accelerating cell-wall loosening in the ER. However, further investigation is needed to test this hypothesis. 

Moreover, stipe elongation requires not only the extension of cell walls, but also the expansion of cell membranes, the increase of cell cytoplasm, and the polar growth of mycelia, as well as probably more processes. Several studies have also shown that ROS regulates root and pollen tube development (elongation) by activating Ca^2+^ and other ion channels in plants, which indicated that ROS- mediated cell elongation is a complex biological process [53,54,55]. Further studies will be needed in the future to reveal the function of different ROS molecular signals in regulating mushroom stipe elongation in more depth.

## 5. Conclusions

In summary, our results demonstrate that the interplay between NADPH oxidase and Mn-type SODs plays a critical role in controlling the distribution of two main ROS (O_2_^−^ and H_2_O_2_) which regulate the gradient elongation of the mushroom stipe. Our findings not only provide insight into the mechanism of mushroom stipe elongation, but also highlight a potential target gene, manipulation of which may benefit mushroom breeding. 

## Data Availability

Data are contained within the article or Supplementary Materials.

## Supplementary Materials

The following supporting information can be downloaded at: https://www.mdpi.com/article/10.3390/cells11121896/s1, Figure S1: The vector maps of the overexpression and RNAi plasmids constructed in this study. (a,b). The overexpression and RNAi plasmids of *FfNoxA*. (c,d) The overexpression and RNAi plasmids of *FfMnSOD1*. (e,f) The overexpression and RNAi plasmids of *FfMnSOD2*. “Hyg” represents the *hygromycin B phosphotransferase* gene, “Ab GPD promoter” represents the *GAPDH* promoter of *A. bisporus*, “Ff GPD promoter” represents the *FfGAPDH* promoter, “Ff GPD Intron” represents the first intron of *FfGAPDH*. The restriction enzyme cutting sites are shown in the maps. Figure S2: Different growth stages of a *F. filiformis* fruiting body. The fruiting body development beginning from (a) the primordia stage (PR) followed by (b) the differentiation stage (DI) and (c) the elongation stage (EL) and maturation stage (MA). Table S1: Primers used in PCR. Table S2: Primers used in real-time quantitative RT-PCR. Table S3: Bio-informatics analysis of amino acid sequences. Table S4: Correlation analysis among gene expression, O_2_^−^ concentration, and stipe elongation rate. Table S5: Detection of the insertion sites of the *FfNoxA* OE and RNAi transformants. Table S6: Detection of the insertion sites of the *FfMnSOD1* OE and RNAi transformants. Table S7: Detection of the insertion sites of the *FfMnSOD2* OE and RNAi transformants.
