# Supplementary material for: Reactive Oxygen Species Distribution Involved in Stipe Gradient Elongation in the Mushroom Flammulina filiformis
Source: Cells. 2022 Jun 11;11(12):1896. doi: 10.3390/cells11121896 (PMC9221348; doi:10.3390/cells11121896)
Supplement: Supplementary file 1 [file cells-11-01896-s001.zip › Table S1.pdf]

Table S1 Primers used in PCR

Gene	Forward sequence（5'-3'）	Reverse sequence（5'-3'）
FfNoxA	TGTCTGGATACGCTCTTTTC	ACACTTGGAGCCCTATAGAA
FfNoxB	GTTGTCATAGTTTCGTCCCT	GTCATCACAGCTAGAAGCAT
FfNoxR	ACACGTTGATGCCCTAATAA	ATACAAATCTGAAGCCCGAG
FfRac1	ACAGTAGTCGGATACCAACA	CAAAGGGAGAAAAGACGGTT
FfBem1	TTACTTTTTGCCTTCACGAC	CAGAAACAAGCACTCAACTT
FfCdc24	TAGTGGATGTCCTCGTCTTT	GGAAACAAGTGGAATGAGTG
FfMnSOD1	TCATCAATCCGTTCTTTCGT	CTCATCATTGGAGTCTTCGT
FfMnSOD2	AGGCTAAAGTGAACCTCTTC	TATACTCTGGCTGTACTCGT
FfCuZnSOD1	AGCCACGTTTCAAGAGATAA	TATTGTCAGGAGGTTTACGC
FfCuZnSOD2	GCTGACTTCGATTTCCAAAC	CATCAAATCGAACACTGCTG
FfCuZnSOD3	CCTTTACATTGGCTACCCTT	ATACCGTGATTTACACTCCG
