# Supplementary material for: Reactive Oxygen Species Distribution Involved in Stipe Gradient Elongation in the Mushroom Flammulina filiformis
Source: Cells. 2022 Jun 11;11(12):1896. doi: 10.3390/cells11121896 (PMC9221348; doi:10.3390/cells11121896)
Supplement: Supplementary file 1 [file cells-11-01896-s001.zip › Table S2.pdf]

Table S2 Primers used in real-time quantitative RT-PCR

Gene	Forward sequence（5'-3'）	Reverse sequence（5'-3'）
FfNoxA	GGTCGTCCTGACTGGATGACTATCTA	GTGTTGCGTAGTTGCCTCCTTGA
FfNoxB	CAGGACACGCAGAACAGGATTGAG	GCCACAGAAGAACACGCCAACA
FfNoxR	CTTCACTACATTCACAGGCGTCACT	TGTCGTCAGGCTTCGGCAGAA
FfRac1	GGGAAGACCTGCCTACTCATTTCG	TCATAATCCTCCTGTCCAGCCGTAT
FfBem1	ACAGGATGGCGGAGCATGGTA	GGCGGAGGGATGTTGAATGAAGAT
FfCdc24	GCCAACTACACCAACGCCTCT	AATGAGCGAGTCCAATAGTAACGGATA
FfMnSOD1	TCAGCATTCGGCAGCCTTGATAAC	ATCCTGGTTGGCGGTGGTAACA
FfMnSOD2	CTGCCTTACGCCTACAATGCTCTC	CGTTGACATAGGTCTGATGGTGCTT
FfCuZnSOD1	TCGTAACTCTGTTGAGCATTGGATTCT	GTGAGGTCTCCGAATTGATGGATATGG
FfCuZnSOD2	GGGACGGATGACTTGGGTAAA	ACACTGCTGTTCGCTCCAATTAC
FfCuZnSOD3	CTACCACATTCACCAGGCAGG	CCAGAGCAATCAAATGGTCCC
FfGAPDH	CCTCTGCTCACTTGAAGGGT	GCGTTGGAGATGACTTTGAA
FfRas	TCAATGCGACGAGTAAAGAGAGG	CATAGGTCCCACATCTACATTTCG
