# Supplementary material for: Reactive Oxygen Species Distribution Involved in Stipe Gradient Elongation in the Mushroom Flammulina filiformis
Source: Cells. 2022 Jun 11;11(12):1896. doi: 10.3390/cells11121896 (PMC9221348; doi:10.3390/cells11121896)
Supplement: Supplementary file 1 [file cells-11-01896-s001.zip › Table S4.pdf]

Table S4 Correlation analysis among gene expression, O_2^- concentration and stipe elongation rate

	elongation rate	O_2^- concerntration	*FfNoxA*	*FfNoxB*	*FfNoxR*	*FfRac1*	*FfBem1*	*FfCdc24*
elongation rate	1.00	0.99	0.99	1.00	0.98	0.95	0.98	1.00
O_2^- concerntration		1.00	0.95	1.00	0.94	0.99	1.00	0.98
FfNoxA			1.00	0.98	1.00	0.89	0.93	0.99
FfNoxB				1.00	0.97	0.97	0.99	1.00
FfNoxR					1.00	0.88	0.92	0.99
FfRac1						1.00	1.00	0.94
FfBem1							1.00	0.97
FfCdc24								1.00
