# Supplementary material for: Reactive Oxygen Species Distribution Involved in Stipe Gradient Elongation in the Mushroom Flammulina filiformis
Source: Cells. 2022 Jun 11;11(12):1896. doi: 10.3390/cells11121896 (PMC9221348; doi:10.3390/cells11121896)
Supplement: Supplementary file 1 [file cells-11-01896-s001.zip › Table S5.pdf]

Table S5 Detection of the insertion sites of the *FfNoxA* OE and RNAi transformants

Transformants	The reads at the joint regions of Genome and Vector sequence	The position of plasmid	The position of genome
FfNoxA$^{OE\#1}$	>ST-E00600:234:H7T7CALXX:2:1170:11984:36276 1:N:0:CCTCCTGA CTTCTATCAATAAAATTTCTAATTCCTAAAACCAA AATCCAGTACTAAAATCCAGATCCCCCGAATTAAT TCGtcgcgatcttggccaggtgttcgtctctatgttacgcgagagttttgcgg ctctcacaactgttgtaggatacgtag	LB	Fv01-10 scaffold16: 285010-285086 bp
	>ST-E00600:234:H7T7CALXX:2:2350:19949:16407 1:N:0:CCTCCTGA CGCAGCCTGAATGGCGAATGCTAGAGCAGCTTGA GCTTGGATCAGATTGTCGTTTCCCGCCTTCAGTTTA AACTATCAGTGaggatcgggccgaatactaataacatgggcattcct cgtaataatttaaaatacagggcatcacccacg	RB	Fv01-10 scaffold16: 284960-284892 bp
FfNoxA$^{OE\#1}$	>ST-E00600:234:H7T7CALXX:2:1326:24433:25019 2:N:0:CCGTGAGA GCAGGGTCGATGCGACGCAATCGTCCGATCCGGA GCCGGGACTGTCGGGCGTACACAAATCGCCCGCA GAAGCGCGGccgcccccgccaccgcccaagtccgcacggcagtcgc gtctctctcgacagctccatatcgaggaggaggacg	LB	Fv01-N scaffold111：1079604-1079676 bp
	>ST-E00600:234:H7T7CALXX:2:2356:16351:14591 2:N:0:CCGTGAGA CTGAATGGCGAATGCTAGAGCAGCTTGAGCTTGG ATCAGATTGTCGTTTCCCGCCTTCAGTTTAAACTAT CAGTGTTTGACTGGGCTGGTCTCAAAAACAACTT TTGGActcctgttgaaacctccgcctctaacacaatatcctccagc	RB	Fv01-N scaffold111：1078401-1078441 bp
FfNoxA$^{RNAi\#1}$	>ST-E00600:302:H7W5HALXX:1:2562:8196:34006 1:N:0:CCTAATCC ATCAGAGCTTGGTTGACGGCAATTTCGATGATGCA GCTTGGGCGCAGGGTCGATGCGACGCAATCGTCC GATCCGGAgccaccgccgcgaccacctcagatgactgaggagggaca ttgggtgccgccgcctcaacagccgtatgggatg	LB	Fv01-10 scaffold5: 348281-348209 bp
	>ST-E00600:302:H7W5HALXX:1:2216:27534:2644 1:N:0:CCTAATCC AACAGTTGCGCAGCCTGAATGGCGAATGCTAGAG CAGCTTGAGCTTGGATCAGATTGTCGTTTCCCGGC TTCAGTTTAAACTAgctgaggcagcatgtgcggcggcattccagg aggaatcggcagcgcacccctcgagggcgtcgtcgt	RB	Fv01-10 scaffold5: 348346-348412 bp
FfNoxA$^{RNAi\#2}$	>ST-E00600:234:H7T7CALXX:2:2301:10800:32503 1:N:0:CGACTGGA GGCTGTGTAGAAGTACTCGCCGATAGTGGAAACC GACGCCCCAGCACTCGTCCGAGGGCAAAGGAAT Agaatggcggaagcctaccaggataatatacatcactatggtgagaaatag aagcgctgatctcctggcacagttgggggaaa	LB	Fv01-10 scaffold7: 899522-899441 bp
	>ST-E00600:234:H7T7CALXX:2:1610:27263:4304 1:N:0:CGACTGGA AATGGCGAATGCTAGAGCAGCTTGAGCTTGGATC AGATTGTCGTTTCCCGCCTTCAGTTTAAACTATCA GTGTTTgacaataaccactgggcttcctctagttcgcagtcgtctttgtgca gtgttgagcctggaacatccgaggatgta	RB	Fv01-10 scaffold7: 899573-899647 bp

Note. Capital letters indicate sequences that match the genome, lowercase letters represent sequences that match the plasmid. LB means the LB T-DNA repeat region of plasmid, RB means the RB T-DNA repeat region of plasmid.
