# Supplementary material for: Reactive Oxygen Species Distribution Involved in Stipe Gradient Elongation in the Mushroom Flammulina filiformis
Source: Cells. 2022 Jun 11;11(12):1896. doi: 10.3390/cells11121896 (PMC9221348; doi:10.3390/cells11121896)
Supplement: Supplementary file 1 [file cells-11-01896-s001.zip › Table S6.pdf]

Table S6 Detection of the insertion sites of the *FfMnSOD1* OE and RNAi transformants

Transformants	The reads at the joint regions of Genome and Vector sequence	The position of plasmid	The position of genome
FfMnSOD1$^{\text{OE\#1}}$	>ST-E00600:234:H7T7CALXX:1:2138:16414:21402 2:N:0:CAACCACA<br>CAATTTGTTTACACCACAATATATCCTGCCACCAGCCAGCCAACAGCTCCCCGACCGGCAGCTCGGCACAAAATCACCACTCGATaattctccatattggttcgcaggtgtaaatctgcagtaccctgcacttcctgatgcccggtcagc	LB	Fv01-10 scaffold3: 1963921-1963857
	>ST-E00600:234:H7T7CALXX:1:2260:6922:25974 1:N:0:CAACCACA<br>GAAGAGGCCCGCACCGATCGCCCTTCCCAACAGTTGCGCAGCCTGAATGGCGAATGCTAGAGCAGCTTGAGCTTGGATCAGATTGTCGTTTCCCGCCTTCAGTTTAAACTATCAGTGTTTggattgggtagactatcgagcgcgggaggg	RB	Fv01-10 scaffold3: 1963931-1963960
FfMnSOD1$^{\text{RNAi\#1}}$	>ST-E00600:234:H7T7CALXX:1:2211:23891:21574 1:N:0:CATACCAA<br>AAAATCCAGTACTAAAATCCAGATCCCCCGAATTAATTCGGCGTTAATTCAGTACATTAAAAACGTCCGCAATGTGTTATTAAGTTGTCTAAGCGTCAATTTGTTTACattcattgctgaaggcgaggttgtacttttccccgtcaaagc	LB	Fv01-10 scaffold9: 437982-438023 bp
	>ST-E00600:234:H7T7CALXX:1:2465:12662:25488 2:N:0:CATACCAA<br>CAACAGTTGCGCAGCCTGAATGGCGAATGCTAGAGCAGCTTGAGCTTGGATCAGATTGTCGTTTCCCGCCTTCAGTTTAAACTATCAGTGTTTacggcaaactagtgattaccatcacccaggagtctatccatgatctcaactggaaaa	RB	Fv01-10 scaffold9: 437810-437754 bp
FfMnSOD1$^{\text{RNAi\#2}}$	>ST-E00600:234:H7T7CALXX:1:2448:26693:23672 2:N:0:CCGAAGTA<br>ATCCAGATCCCCCGAATTAATTCGGCGTTAATTCAGTACATTAAAAACGTCCGCAATGTGTTATTAAGTTGTCTAAGCGTCAAcaagtcaagtaaactcgtacaaatttcgtacgctctgcctttttcggtgatgtcgctgcttcgcgtt	LB	Fv01-10 scaffold1: 2547426-2547492 bp
	>ST-E00600:234:H7T7CALXX:1:2502:2799:23187 2:N:0:CCGAAGTA<br>CAGCCTGAATGGCGAATGCTAGAGCAGCTTGAGCTTGGATCAGATTGTCGTTTCCCGCCTTCAGTTTAAACTATCAGTGTttgctatggccactggccgtcgaggtcgctctagaaaaacacgagacgcaacgtccgaggatcccaacga	RB	Fv01-10 scaffold1: 2547394-2547325 bp

Note. Capital letters indicate sequences that match the genome, lowercase letters represent sequences that match the plasmid. LB means the LB T-DNA repeat region of plasmid, RB means the RB T-DNA repeat region of plasmid.
