# Supplementary material for: Reactive Oxygen Species Distribution Involved in Stipe Gradient Elongation in the Mushroom Flammulina filiformis
Source: Cells. 2022 Jun 11;11(12):1896. doi: 10.3390/cells11121896 (PMC9221348; doi:10.3390/cells11121896)
Supplement: Supplementary file 1 [file cells-11-01896-s001.zip › Table S7.pdf]

Table S7 Detection of the insertion sites of the *FfMnSOD2* OE and RNAi transformants

Transformants	The reads at the joint regions of Genome and Vector sequence	The position of plasmid	The position of genome
FfMnSOD2[OE#1]	>ST-E00600:298:H7JGHALXX:3:2161:1081:15577 1:N:0:CCTCTATC<br>AACCGACGCCCCAGCACTCGTCCGAGGGCAAAGGAATAGAGTAGATGCCGACCGGGATCTCGAGTTTCTCCATAATtagaggacaaatgaattgatcgattctactgagaggtttggaatgccggtggcgcgtcgattcggcgccagctg	LB	Fv01-10 scaffold8: 568957-568884 bp
	>ST-E00600:298:H7JGHALXX:3:1367:2808:5760 1:N:0:CCTCTATC<br>TTAATCGCCTTGCAGCACATCCCCCTTTCGCCAGCTGGCGTAATAGCGAAGAGGCCCGCACCGATCGCCCagaaacaactgaagaactcaccaatttctctacagaggtctatatcgccgtcaatgcatgttgctgagtacatctaagct	RB	Fv01-10 scaffold8: 569479-569558 bp
FfMnSOD2[OE#2]	>ST-E00600:234:H7T7CALXX:1:1655:28809:20008 2:N:0:CTCAATGA<br>CAGATCCCCCGAATTAATTCGGCGTTAATTCAGTACATTAAAAACGTCCGCAATGTGTTATTAAGTTGTCTAAGCGTCAATTTGTTTACACCcggccgtaacccacctaaaagactgttagtgattatcagtaacagaacagcagcgacg	LB	Fv01-10 scaffold19: 36410-36467 bp
	>ST-E00600:234:H7T7CALXX:1:1604:14940:18693 1:N:0:CTCAATGA<br>CGCAGCCTGAATGGCGAATGCTAGAGCAGCTTGAGCTTGGATCAGATTGTCGTTTCCCGCCTTCAGTTTAAACTATCAGTGTTTGAttggggcccttgggatcccggggaattacacgtcgaaggcccttcaagagaagattgcctggct	RB	Fv01-10 scaffold19: 36365-36302 bp
FfMnSOD2[RNAi#1]	>ST-E00600:298:H7JGHALXX:2:1415:31213:2472 1:N:0:CGACACAC<br>gtttagaaagggatctgtactggttcggacggaggtgactcttggatccaaagtatactcgttaacatggattgactatcgtacagatacctcaagTTGGGTAACGCCAGGGTTTTCCCAGTCACGACGTTGTAAAACGACGGCCAGTGC	LB	Fv01-10 scaffold3: 3043983-3044078 bp
	>ST-E00600:298:H7JGHALXX:2:2273:27136:9220 1:N:0:CGACACAC<br>acagccagagctcgcgtgttggtgtcatagacgacgcaaacaccactcctcaacgtcgttcgcaccttttcgtcgttcgcctgctcgaacAGGATATATTGTGGTGTAAACAAATTGACGCTTAGACAACTTAATAACACATTGCGGAC	RB	Fv01-10 scaffold3: 3048146-3048236 bp

Note. Capital letters indicate sequences that match the genome, lowercase letters represent sequences that match the plasmid. LB means the LB T-DNA repeat region of plasmid, RB means the RB T-DNA repeat region of plasmid.
