# Supplementary figures and images for: Reactive Oxygen Species Distribution Involved in Stipe Gradient Elongation in the Mushroom Flammulina filiformis
Source: Cells. 2022 Jun 11;11(12):1896. doi: 10.3390/cells11121896 (PMC9221348; doi:10.3390/cells11121896)

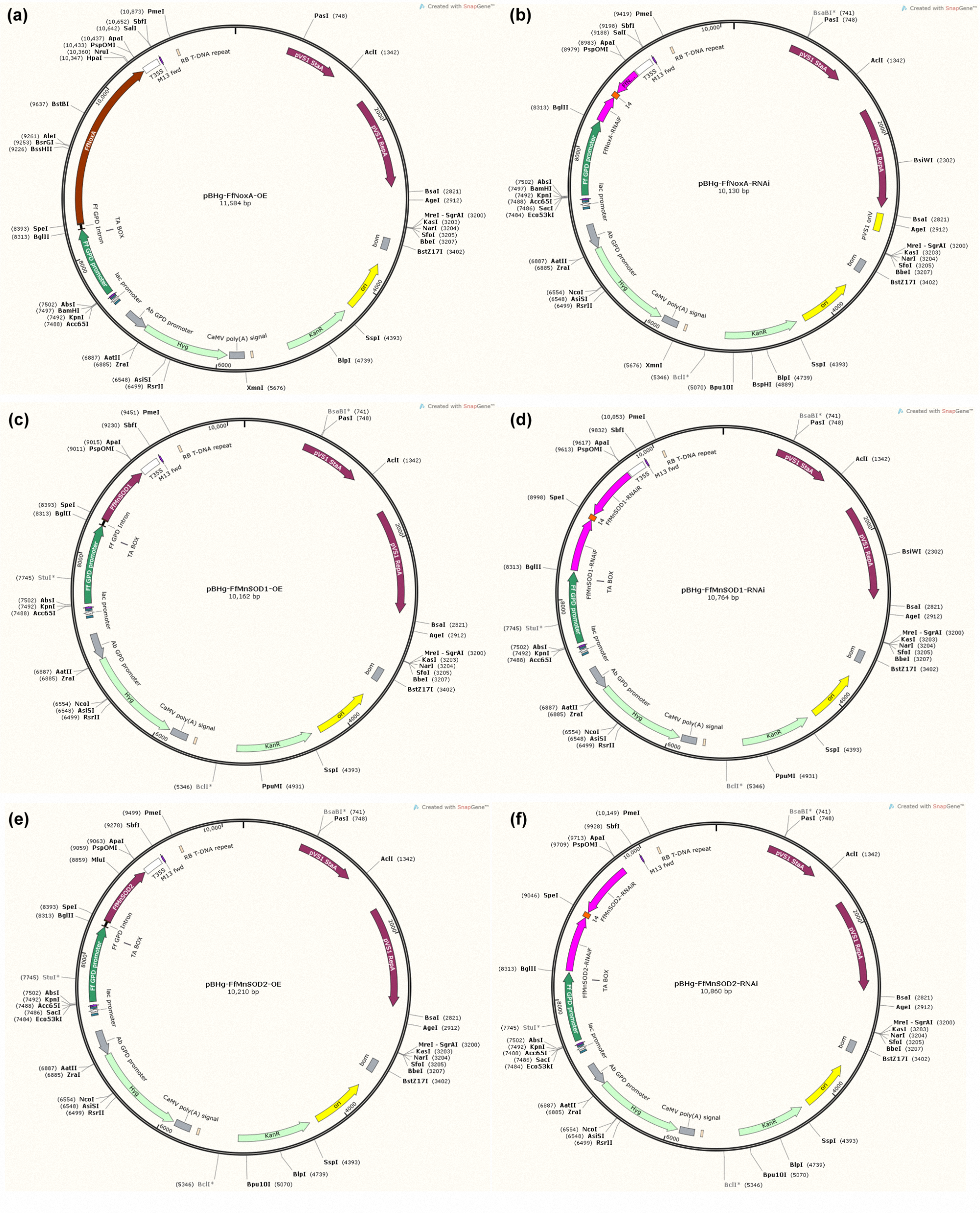

Supplement: Supplementary file 1 [file cells-11-01896-s001.zip › Figure S1.tif]

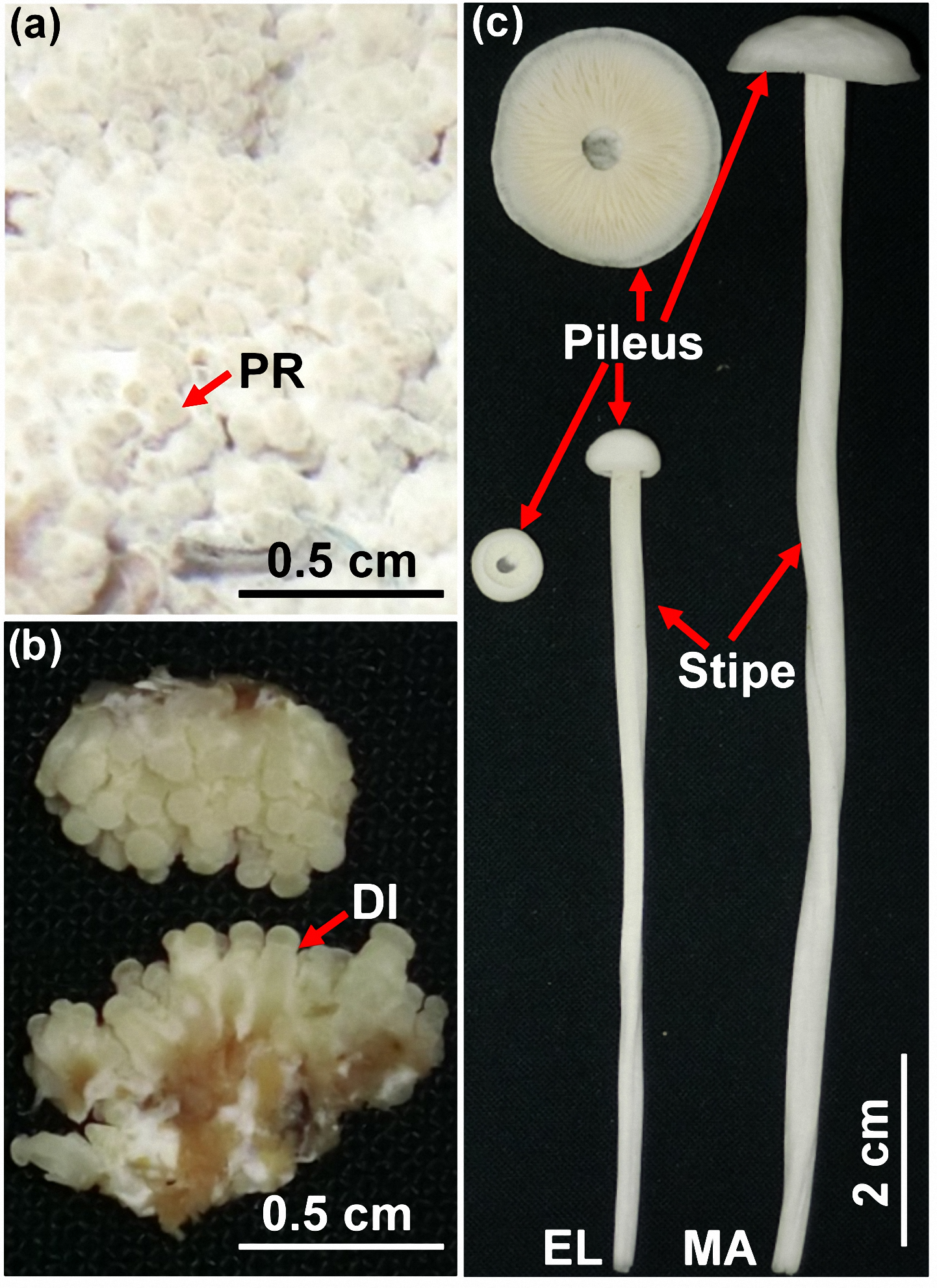

Supplement: Supplementary file 1 [file cells-11-01896-s001.zip › Figure S2.tif]
